# Turnover in Zero-Premium Status Among Health Insurance Marketplace Plans Available to Low-Income Enrollees

**DOI:** 10.1001/jamahealthforum.2022.0674

**Published:** 2022-04-22

**Authors:** Edward Kong, Mark Shepard, Adrianna McIntyre

**Affiliations:** 1 Harvard University, Boston, Massachusetts; 2 Harvard Medical School, Boston, Massachusetts; 3 Harvard Kennedy School, Cambridge, Massachusetts; 4 National Bureau of Economic Research, Cambridge, Massachusetts; 5 Harvard TH Chan School of Public Health, Boston, Massachusetts

## Abstract

**Importance:**

Recent subsidy enhancements in Affordable Care Act (ACA) Marketplaces made many low-income enrolles (below 150% of the federal poverty level [FPL]) eligible for 2 free silver-tier plans. eligible for 2 free silver-tier plans. However, an unintended consequence of this structure is that the identity of which silver plans are free will often “turn over” between years, requiring that enrollees actively initiate premium payment (or lose coverage). The prevalence of this free-plan turnover is not known.

**Objective:**

To measure the prevalence of free-plan turnover in ACA Marketplaces and to estimate how many enrollees below 150% of FPL are likely to be affected.

**Design, Setting, and Participants:**

This observational cross-sectional study used data on plan offerings and premiums in 33 state ACA Marketplaces using HealthCare.gov in 2021 and 2022, along with estimates of county-level enrollee characteristics and plan selection patterns. The enrollment-weighted share of county markets affected by free-plan turnover was quantified, along with the association of turnover with enrollee and market characteristics. Estimates of the number of affected low-income enrollees were calculated using the data plus statistics reported in past research. Data were analyzed from November 21, 2021, to February 28, 2022.

**Results:**

This study found that turnover of zero-premium plans was quite common, with 93% of HealthCare.gov counties (weighted by enrollment) experiencing at least 1 zero-premium plan in 2021 turning over to nonfree in 2022; 84% of counties experienced turnover of all $0 silver plans from 2021 to 2022. This turnover affected an estimated 1.36 million people with incomes below 150% of FPL. Turnover was more common in counties with a higher share of non-White enrollees, in Medicaid nonexpansion states, in counties with more carriers, and in counties with changes in the number of offered plans.

**Conclusions and Relevance:**

The findings of this cross-sectional study suggest that owing to the prevalence of zero-premium plan turnover, many low-income ACA enrollees faced elevated risk of disenrollment at the start of 2022. Outreach to affected enrollees and other actions to encourage coverage retention and midyear reenrollment could help mitigate coverage losses.

## Key Points

**Question:**

How often did silver-tier marketplace plans that were free to low-income enrollees in 2021 become nonfree in 2022—putting enrollees at risk of termination if they did not switch plans or initiate premium payments?

**Findings:**

In this observational cross-sectional study of plan and enrollment data from 33 HealthCare.gov states, turnover of zero-premium plans occurred in 93% of counties (weighted by enrollment) from 2021 to 2022.

**Meaning:**

In this study, the risk of coverage loss was elevated among a large number of enrollees experiencing free-plan turnover at the start of 2022; outreach or other steps could help mitigate terminations or reenroll affected people.

## Teaser

This observational cross-sectional study measures the prevalence of free-plan turnover in the Affordable Care Act Marketplaces and estimates how many enrollees below 150% of the federal poverty level are likely to be affected.

## Introduction

The American Rescue Plan Act (ARPA), COVID-19 relief legislation passed in March 2021, substantially increased the subsidies available to make coverage on the health insurance marketplaces more affordable. Under the new subsidy rules, households with income below 150% of the federal poverty level (FPL) generally qualify for two plans with $0 premiums on the “silver” tier—the benchmark (second lowest-cost silver) plan—plus the lowest-cost plan in the tier.^1^ In some circumstances, these zero-dollar silver plans are available even farther up the income scale.^2^ This boost to financial assistance is temporary, expiring at the end of 2022 unless Congress passes new legislation.

Prior to ARPA, where zero-dollar plans were available to marketplace enrollees, they were plans in the less-generous “bronze” tier.^3^ Bronze plans are particularly unattractive to enrollees at lower income levels; households below 250% FPL qualify for additional financial assistance to offset out-of-pocket costs when seeking care, but these “cost-sharing reductions” are only available in silver plans.^4^

Although zero-dollar premiums may facilitate higher enrollment, which 2 silver plans are $0 can change year over year as insurers compete on price.^5^ Consequently, automatically renewed enrollees could have gone from having free coverage in December 2021 to owing a premium in January 2022 simply by staying enrolled in their health plan as it was undercut by competitors.^6^

Although a good number of returning enrollees appear to shop during the open enrollment period and switch plans, about 40% of enrollees, on average across all income levels, were automatically renewed into their plans over the past 3 years.^7^ This is consistent with recent evidence documenting considerable inertia in the individual market.^8,9,10^ If an enrollee autorenewed into a plan that changed from zero premium to positive premium and failed to pay their new premiums—because they were not aware of the change or did not set up an automatic payment mechanism when they initially elected their plan, for example—their coverage could be terminated.

This turnover in zero-premium plans could lead to meaningful spikes in terminations unless steps are taken to retain affected enrollees.^11^ We characterize how commonplace these transitions were for 2021 to 2022 renewals in states using the federally facilitated marketplace platform, HealthCare.gov, and assess characteristics of affected counties.

## Methods

### Data and Sample

For states using HealthCare.gov, we obtained county-level plan premium data from the Health Insurance Exchange Public Use Files (2021-2022) and 2021 enrollment data from the Marketplace Open Enrollment Period Public Use Files, both published by the Centers for Medicare & Medicaid Services (CMS).^12,13^ This study was deemed not human participants research and exempt from review by the Harvard Longwood Campus institutional review board because all data are county level and publicly available.

### Statistical Analysis

For each county, we identified the 2 lowest-cost silver plans. We also identified cases where 1 or both of these 2 plans would not be $0 for enrollees below 150% FPL owing to benefit structure.^14^ Using data on open enrollment for the 2021 plan year, we estimated the share of enrollees in the affected 100% to 150% FPL group who would experience (1) at least 1, or (2) all available $0 plans ceasing to be zero premium in 2022 because they were no longer among the 2 lowest-cost offerings.

### Characteristics Associated With Affected Counties

We used bivariate regression to assess features associated with counties experiencing turnover in zero-premium silver plans. The characteristics we considered included expansion status, the race and ethnicity of enrollees, the number of carriers offering plans in the county, and changes to the number of plans offered in the county.

Enrollment data by race and ethnicity were derived from individual state-based marketplaces, which collect self-reported race (eg, Asian, Black, White, other, unknown) and ethnicity (Hispanic, Non-Hispanic, or unknown ethnicity) as separate, independent questions.^15^ We defined “non-White share” as 1 minus the share selecting “Race = White” in each county. This metric included individuals who selected “other” race, multiple races, or where race was not reported. We examined how the likelihood of living in an affected county varied with county shares of each race and ethnicity. County-subgroup combinations with fewer than 10 individuals were missing in the data.

For ease of interpretation, we report quartile estimates (with 95% CIs); bivariate regression results are available in eTable 3 in the Supplement.

### Number of Enrollees Experiencing Transitions

The available data are at the county level and do not report plan-specific enrollment by income level, so they do not permit direct observation of the number of enrollees electing zero-premium silver plans. To generate a back-of-the-envelope estimate of the number of enrollees affected by turnover in zero-premium status, we assumed that the share of enrollees in the 100% to 150% FPL band that chose a silver plan was 85%. It has been estimated that 89% of enrollees in this income band selected silver-tier plans between 2014 and 2016.^16^ We used a slightly lower number because more recent evidence suggests that preference for silver-tier plans fell very slightly (by several percentage points among households with incomes 100%-150% FPL) after the introduction of “silver loading” in some marketplaces made bronze-tier plans relatively cheaper.^17^ Of the expected silver enrollees, we assumed that the share choosing zero-premium silver plans was 60% in counties with 2 free plans and 30% in counties with 1 free plan, consistent with evidence in the literature.^18^

All analyses were conducted using Stata statistical software (version 17; StataCorp, LLC); significance was determined at the *P* < .05 level using 2-sided tests. Data were analyzed from November 21, 2021, to February 28, 2022. We followed the Strengthening the Reporting of Observational Studies in Epidemiology (STROBE) reporting guidelines.

## Results

Our data source covers 2447 counties across the 33 states that used HealthCare.gov in 2021 and 2022; information on enrollment in the 100% to 150% FPL group was available for 96% (N = 2351) of these counties. These data represent the enrollment of 3.34 million enrollees in the income group of interest. Our bivariate analyses were restricted to the 2187 counties in 29 states (representing 3.26 million enrollees) where at least 1 $0 plan was available in 2021.

For some analyses stratifying by race and ethnicity, the number of counties in our sample was smaller than 2187 because county-subgroup combinations with fewer than 10 individuals were missing in the data. Table 1 reports descriptive statistics for the counties included in our analysis. Unless otherwise indicated, enrollee characteristics represent enrollees at all income levels; data on metal level and self-reported race and ethnicity are not stratified by income.

**Table 1.** Characteristics of Counties Included in Analysis

Characteristic	Mean (SD)
County enrollee characteristics^a^	
Share of enrollees 100%-150% FPL	0.30 (0.17)
Share of enrollees in silver plans	0.52 (0.17)
Share of counties with at least one $0 plan in 2021	0.92 (0.27)
Share of counties with at least one $0 plan in 2022	0.88 (0.33)
Share of Asian enrollees^b^^,^^c^	0.04 (0.04)
Share of Black enrollees^b^^,^^c^	0.06 (0.09)
Share of White enrollees^c^	0.60 (0.18)
Share of Hispanic enrollees^b^	0.07 (0.10)
County market characteristics, median (IQR) [range]^d^	
No. of enrollees with incomes 100%-150% FPL	190 (70-575) [0-379 208]
No. of participating issuers	2 (2-3) [1-10]
No. of silver plans available	9 (5-21) [2-70]

Abbreviation: FPL, federal poverty level.

^a^ We reported mean (SD) for enrollee characteristics because they are generally normally distributed. Data are from 2021 unless otherwise noted, and include N = 2447 counties.

^b^ Counties with the lowest shares of Asian, Black, and Hispanic enrollee shares are censored because data are missing for cells where 0 < N < 11.

^c^ A large share of enrollees reported “other” race or did not report race; as a result, the shares of Asian, Black, and White enrollees did not approach 1 when summed.

^d^ We reported median (IQR) and overall range for market characteristics because they are generally not normally distributed.

**Figure 1.**
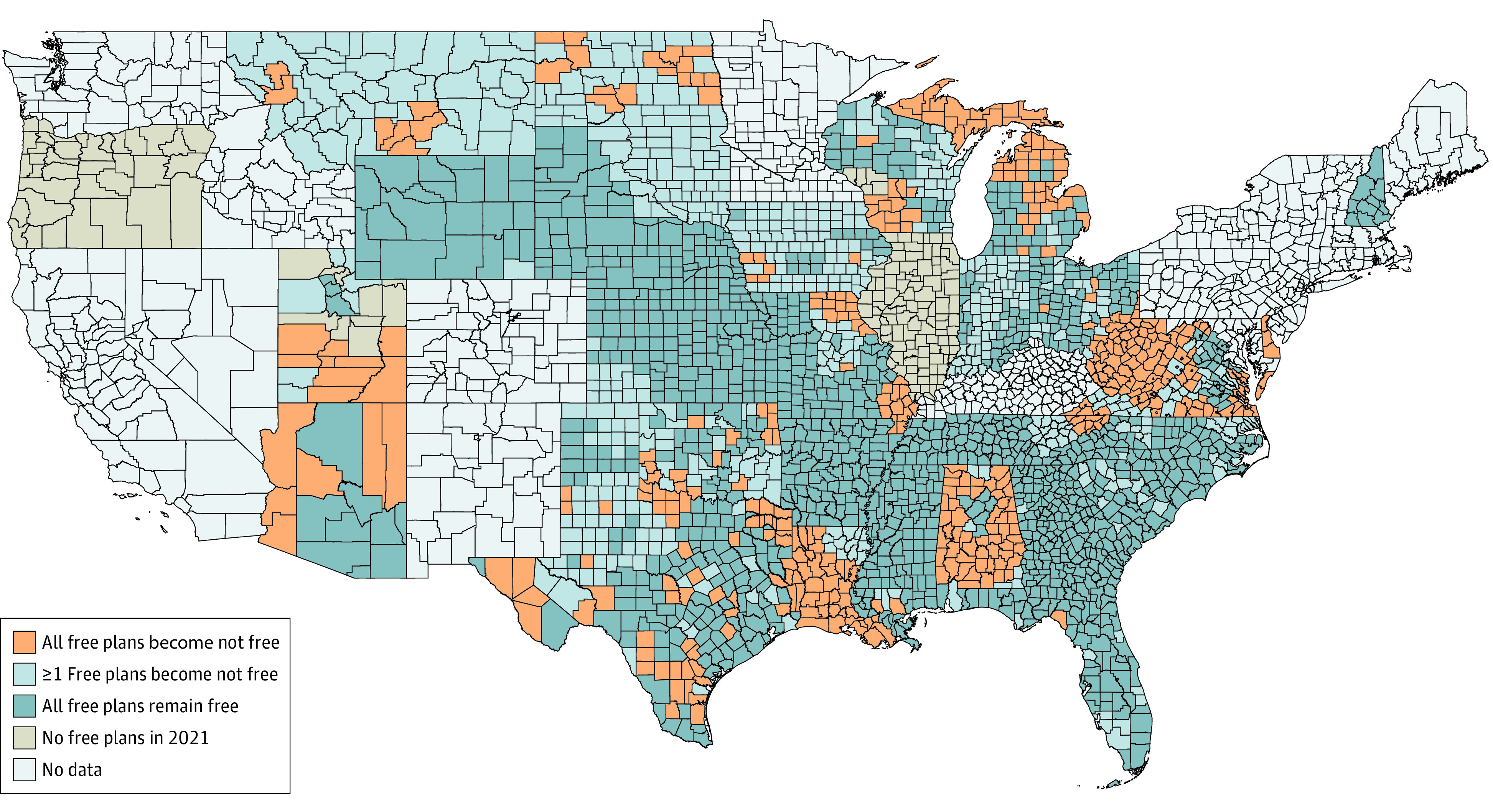
Counties Where Silver-Tier $0 Plans in 2021 Become Positive-Premium Plans in 2022

Owing to a few large states (eg, Texas and Florida), states that have not expanded their Medicaid programs under the ACA accounted for 78% of total marketplace enrollment. However, they accounted for 91% enrollment among individuals in the 100% to 150% FPL income group (eTable 1 in the Supplement). On average, the 100% to 150% FPL income group represented slightly less than one-third of county enrollment (Table 1), but this figure was 42% in nonexpansion counties, compared with only 16% of the average expansion county. Overall, 93% of enrollees in the 100% to 150% FPL group lived in counties where at least 1 silver plan transitioned from $0 in 2021 to a positive premium in 2022 (Table 2); for 84% of enrollees in this income group, all 2021 $0 silver plans in their county transitioned to positive premiums in 2022. These counties are plotted in Figure 1. The share of enrollees affected was even greater in counties where at least 1 $0 silver plan was offered in 2021 (Table 2). eTable 2 in the Supplement reports these estimates not weighted by enrollment.

**Table 2.** Shares of Counties Affected and Select Associated County Characteristics

Characteristic	No.	At least 1 $0 plan became positive-premium^a^	All $0 plans became positive-premium^a^
All HealthCare.gov counties^b^	2351	0.93 (0.92-0.94)	0.84 (0.83-0.86)
All HealthCare.gov counties offering at least 1 $0 silver plan in 2021^b^	2187	0.96 (0.95-0.96)	0.86 (0.85-0.88)
Factors correlated with county experiencing $0 to positive-premium transitions^c^			
Share of non-White enrollees, quartiles^d^			
Q1 (0.05%-0.27%)	543	0.72 (0.65-0.80)	0.45 (0.37-0.53)
Q2 (0.27%-0.38%)	543	0.84 (0.79-0.89)	0.62 (0.55-0.70)
Q3 (0.38%-0.53%)	543	0.93 (0.91-0.96)	0.75 (0.68-0.82)
Q4 (0.53%-0.98%)	543	0.98 (0.97-0.99)	0.92 (0.87-0.97)
Share of county marketplace enrollees in 100%-150% FPL income group, quartiles^d^			
Q1 (0.03-0.17)	547	0.82 (0.75-0.88)	0.58 (0.46-0.71)
Q2 (0.16-0.31)	547	0.87 (0.82-0.92)	0.64 (0.53-0.74)
Q3 (0.31-0.43)	547	0.94 (0.92-0.96)	0.80 (0.74-0.86)
Q4 (0.43-0.83)	546	0.98 (0.96-0.99)	0.92 (0.86-0.97)
No. of carriers offering plans in the county			
1	207	0.45 (0.30-0.60)	0.28 (0.13-0.43)
2-4	1728	0.94 (0.93-0.96)	0.76 (0.69-0.83)
≥5	252	1.00 (1.0-1.0)	0.97 (0.94-1.0)
Medicaid expansion status			
Expansion state		0.85 (0.80-0.90)	0.68 (0.59-0.77)
Nonexpansion state		0.97 (0.95-0.98)	0.88 (0.83-0.93)

^a^ Results show 95% CIs using robust standard errors.

^b^ Table reports share of counties weighted by number of enrollees with income between 100% and 150% of federal poverty level in 2021, limited to 2351 counties with enrollment data or 2187 counties with enrollment data and at least 1 $0 silver plan in 2021.

^c^ Excludes counties where the 2 cheapest silver plans covered benefits in addition to the mandated set of essential health benefits, and hence had premiums higher than $0.

^d^ In bivariate regression analysis, each of these factors was significantly correlated with $0 to positive-premium transitions; quartiles are presented for ease of interpretation. Analyses of county characteristics are limited to N = 2187 counties with at least 1 $0 plan in 2021 and enrollment data for the 100% to 150% FPL group silver plan enrollment data available. Data on share of non-White enrollees were available for 2172 of these counties, and data on the share of enrollees between 100% to 150% FPL were available for 2187 counties.

Counties with a greater share of non-White individuals were significantly more likely to be affected. Enrollees in the top quartile of county non-White share were affected 98% of the time—26 percentage points more than the bottom quartile of non-White share (72%). Counties in the top quartile of Black enrollee share had a 90% chance of having all $0 plans become positive-premium plans, compared with only a 57% chance for the bottom quartile of Black enrollee share. We also found that counties in the top quartile of Hispanic share had a 92% chance of having all $0 plans become positive-premium plans, compared with a 75% chance for the bottom quartile. Results were similar, though not as stark, if the outcome variable was defined as counties with at least 1 $0 plan becoming positive-premium plans (estimates are available in eTable 4 in the Supplement). These results demonstrate that turnover disproportionately affected counties with high shares of Black and Hispanic enrollees.

**Figure 2.**
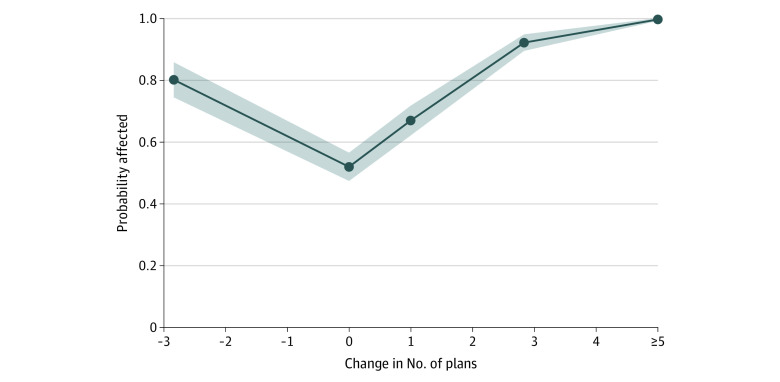
Share of Counties Affected and Change in Number of Available Plans, 2021 to 2022 Points represent within-bin averages for counties adding between −9 and −1 plans, 0 plans, 1 plan, or 2 to 4 plans, with a separate point at *x* = 5 for counties adding 5 or more plans. Shaded region represents 95% CI based on robust standard errors.

Enrollees in counties with more competing carriers were also more likely to be affected because cheaper plans were more likely to be replaced between years (eFigure 3 in the Supplement). We also examined the role of plan entrance and exit directly. Most counties (70%) experienced plan entry between 2021 to 2022; in 21% of counties there was no change in plans, and 9% of counties experienced a decline in plans (eFigure 2 in the Supplement). As shown in Figure 2, counties with no change in the number of plans were least likely to be affected, whereas counties with increases or decreases in the number of plans tended to see turnover in the set of zero-premium plans. Approximately one-third of counties added 5 or more plans for the 2022 plan year and were virtually guaranteed to be affected by turnover in zero-premium plans (eFigure 1 in the Supplement).

In 2021, there were 3.26 million marketplace enrollees with incomes between 100% to 150% FPL in the 2187 counties that had at least 1 zero-premium silver plan available. Based on the assumptions described herein, we estimated that 2.77 million (3.26 million × 85%) of these enrollees selected silver plans (at any price) in 2021. We also estimated that 1.51 million selected zero-premium silver plans (2.77 × 60% in counties where 2 zero-premium plans were offered and × 30% in counties where 1 zero-premium plan was offered in 2021). Of these, we estimated that 1.36 million enrollees (90%) would have had a 0$ premium in 2021 that turned into a positive premium if they maintained enrollment into 2022. Because nonexpansion states were more likely to be affected and comprise a larger share of individuals in the 100% to 150% FPL income group, they accounted for 92.4% of the enrollees whose zero-premium plan in 2021 took on positive premiums in 2022.

## Discussion

Most HealthCare.gov enrollees with incomes between 100% and 150% FPL resided in a county where at least 1 silver plan that was $0 in 2021 became a positive-premium plan in 2022. Prior work suggests this kind of zero-to-positive transition, paired with autorenewal and enrollee inertia, can lead to coverage loss.^8^ This is a potentially significant unintended consequence of a system with a mixture of $0 and positive-premium plans.

Our back-of-the-envelope estimates suggest that 1.36 million enrollees experienced zero-to-positive transitions at the start of 2022. This number likely represents a lower bound, for 2 key reasons. First, in some counties, zero-premium silver plans were available to households at income levels above 150% FPL.^2^ Second, zero-premium bronze plans were even more widely available than zero-premium silver plans—79 percent of enrollees had access to a $0 bronze plan in 2021 after the ARPA subsidy boost went into effect—and there was likely similar volatility in that tier.^19^ Modeling transitions under those circumstances is more complicated with available data and is beyond the scope of this study.

Under ACA rules, people continuously enrolled in their plans are permitted 3 months of payment delinquency before their coverage is terminated. This grace period extends to enrollees who are automatically renewed into a plan that experienced premium changes.^20^ Accordingly, enrollees who were automatically renewed into plans that experienced turnover in zero-premium status would have to pay all newly owed premiums by the end of March 2022 to remain enrolled; failure to make these payments would result in coverage termination.

New rules allow low-income enrollees to switch plans or reenter coverage midyear, which could help mitigate coverage loss—but only if enrollees understand changes to their enrollment status.^21^ Enrollees with higher incomes cannot reenter marketplace coverage midyear without a qualifying life event. Even temporary gaps in coverage can lead to adverse outcomes, including skipped care (if a person knows they lost coverage) or potential surprise medical bills (if they do not).^22^ Those rules are also limited to households below 150% FPL who have access to a zero-premium silver plan and will cease to apply if the ARPA subsidy enhancement is permitted to lapse.

As of now, the subsidy boost is scheduled to conclude at the end of 2022, with subsidies reverting to pre-ARPA levels in January 2023. If this happens, nearly all current zero-premium silver plans will take on positive premiums, as will many zero-premium bronze plans, presenting meaningful barriers to retention.

Regulators may need to consider steps to mitigate unintended terminations, including direct outreach to affected enrollees. The ability for enrollees below 150% FPL to easily reenter coverage midyear presents an opportunity to minimize gaps in coverage. In the longer term, policy makers may need to consider automatic retention policies for enrollees who miss premium payments but remain eligible for zero-premium plans, transitioning people to such plans in the event of payment lapses.^8^ If the ARPA subsidy enhancement is not extended, proactive outreach will be imperative to mitigate potential coverage losses at the start of 2023.

### Limitations

This study has several limitations. Data were only available at the county level, stratified by income band or metal tier (eg, bronze vs silver plans), but not both features simultaneously. We also could not observe specific plan selections of enrollees in this income group. This means we could not directly observe the number of enrollees with incomes between 100% and 150% FPL who elected silver plans. The data published by CMS are also limited to enrollments during the open enrollment period on marketplaces that use the federally facilitated HealthCare.gov platform. Experiences of midyear enrollees and enrollees on state-based marketplaces could be different.

There are also several reasons to exercise caution in interpreting our estimate of the number of enrollees affected by zero-premium turnover. First, our estimate of total enrollees in the 100% to 150% income band was based on open enrollment figures published by CMS; if midyear attrition outpaced midyear enrollment, this may be an overestimate of the total number of enrollees in December 2021. Second, our assumptions about the share of enrollees selecting the lowest- or second lowest-cost silver plans relied on research using data from Covered California; this state-based marketplace requires plan standardization and limits the number of plans a carrier can offer to make comparison shopping easier.^23^ It is possible that these assumptions do not generalize well to HealthCare.gov states, which generally lack these regulations and where consumers have substantially more plans to choose from.^24^

Despite these concerns, we believe that our estimate is reasonable and likely represents a lower bound on the total number of enrollees (across all income levels) affected by zero-premium turnover. As described herein, we limited our analysis to people in the 100% to 150% FPL income band with access to zero-premium silver plans, but these plans are available higher up the income scale in some counties, and similar volatility would plague zero-premium bronze plans. In addition, midyear enrollment in 2021 was bolstered by the introduction of the ARPA subsidy enhancement and related policies; CMS offered a special enrollment period from February through August 2021, during which time over 2.5 million people entered marketplace coverage.^25^

## Conclusions

The ARPA meaningfully expanded affordability of coverage on state marketplaces by expanding financial assistance available to enrollees, and specifically made zero-premium silver-tier plans available for certain low-income households. Our analysis highlights the fact that the specific silver plans that are free are likely to change between years as carriers adjust premiums. Unless free-plan enrollees in 2021 actively switched plans at the start of 2022, most will owe positive premiums that—if not actively paid—could lead to termination of coverage. We estimate that at least 1.36 million low-income enrollees likely faced this situation at the start of 2022 and (based on prior work) are therefore at high risk of premium lapsing and coverage termination. Outreach could help mitigate terminations and encourage lapsed enrollees to reenroll midyear, something that is allowed for most enrollees below 150% FPL. However, challenges with zero-to-positive premium transitions will be even greater in 2023 if ARPA's subsidy increases are not extended.

## Notes

Supplement. eTable 1. Share of enrollment by Medicaid expansion status eTable 2. Shares of counties affected, by share non-White and share of 100%-150% FPL enrollees eTable 3. Bivariate association between zero-premium plan turnover and county race/ethnicity eTable 4. Zero-premium plan turnover by quartile of county race/ethnicity share eFigure 1. Turnover in zero-premium plans is associated with changes in the number of carriers eFigure 2. Turnover in zero-premium plans is associated with changes in the number of plans eFigure 3. Turnover in zero-premium plans is associated with the number of carriers in 2021
