# Error in Figure 1

**DOI:** 10.1001/jamahealthforum.2022.1616

**Published:** 2022-05-27

In the article titled “Turnover in Zero-Premium Status Among Health Insurance Marketplace Plans Available to Low-Income Enrollees”^1^ published on April 22, 2022 in *JAMA Health Forum*, there was an error in the Key for Figure 1. The orange and dark blue colors were incorrectly switched. This article was corrected online.
