# Supplementary material for: Turnover in Zero-Premium Status Among Health Insurance Marketplace Plans Available to Low-Income Enrollees
Source: JAMA Health Forum. 2022 Apr 22;3(4):e220674. doi: 10.1001/jamahealthforum.2022.0674 (PMC9034407; doi:10.1001/jamahealthforum.2022.0674)
Supplement: Supplement. — eTable 1. Share of enrollment by Medicaid expansion status eTable 2. Shares of counties affected, by share non-White and share of 100%-150% FPL enrollees eTable 3. Bivariate association between zero-premium plan turnover and county race/ethnicity eTable 4. Zero-premium plan turnover by quartile of county race/ethnicity share eFigure 1. Turnover in zero-premium plans is associated with changes in the number of carriers eFigure 2. Turnover in zero-premium plans is associated with changes in the number of plans eFigure 3. Turnover in zero-premium plans is associated with the number of carriers in 2021 [file jamahealthforum-e220674-s001.pdf]

Supplemental Online Content

Kong E, Shepard M, McIntyre A. Turnover in zero-premium status among health insurance marketplace plans available to low-income enrollees. *JAMA Health Forum*. 2022;3(4):e220674. doi:10.1001/jamahealthforum.2022.0674

eTable 1: Share of enrollment by Medicaid expansion status

eTable 2: Shares of counties affected, by share non-White and share of 100-150% FPL enrollees

eTable 3: Bivariate association between zero-premium plan turnover and county race/ethnicity

eTable 4: Zero-premium plan turnover by quartile of county race/ethnicity share

eFigure 1: Turnover in zero-premium plans is associated with changes in the number of carriers

eFigure 2: Turnover in zero-premium plans is associated with changes in the number of plans

eFigure 3: Turnover in zero-premium plans is associated with the number of carriers in 2021

This supplemental material has been provided by the authors to give readers additional information about their work.

© 2022 Kong E et al. *JAMA Health Forum.*

Online Appendix:

Turnover in Zero-Premium Status Among Health Insurance Marketplace Plans Available to Low-Income Enrollees

Edward Kong, Mark Shepard, Adrianna McIntyre

Medicaid expansion and total enrollment shares

Enrollment in our sample was concentrated in states that did not expand their Medicaid programs. This was especially true for enrollment in the 100-150% FPL income group (**eTable 1**).

eTable 1: Share of enrollment by Medicaid expansion status

Expansion status	Share of total enrollment	Share of enrollment 100-150%FPL
State expanded	0.22	0.09
State has not expanded	0.78	0.91

© 2022 Kong E et al. *JAMA Health Forum.*

Unweighted results for Table 2

The results we present in the manuscript are weighted by enrollment in the 100-150% FPL income band. This weighting ensures that our findings are not biased by giving sparsely populated rural counties equivalent weight as densely populated urban counties representing more of the affected population. However, we thought it was important to present unweighted estimates for comparison (see **eTable 2** below).

eTable 2: Shares of counties affected, by share non-White and share of 100-150% FPL enrollees

	At least one $0 plan becomes positive-premium	All $0 plans become positive-premium
**All HealthCare.gov Counties (N = 2,351)[1]**	0.75 [0.74–0.77]	0.52 [0.50–0.55]
**All HealthCare.gov Counties offering at least one $0 silver plan in 2021 (N = 2,187)[2]**	0.81 [0.79–0.83]	0.56 [0.54–0.58]
**Factors correlated with county experiencing $0 to positive-premium transitions[3]**		
Share of non-White enrollees (quartiles)		
Q1 (0.04–0.27, N = 543)	0.78 [0.75–0.82]	0.43 [0.39–0.47]
Q2 (0.27–0.38, N = 543)	0.80 [0.77–0.84]	0.52 [0.49–0.57]
Q3 (0.38–0.53, N = 543)	0.80 [0.76–0.83]	0.56 [0.52–0.61]
Q4 (0.53–0.98, N = 543)	0.86 [0.83–0.89]	0.73 [0.69–0.77]
Share of county marketplace enrollees in 100-150% FPL income group (quartiles)		
Q1 (0.03–0.17, N = 547)	0.75 [0.71–0.78]	0.35 [0.31–0.39]
Q2 (0.16–0.31, N = 547)	0.80 [0.77–0.83]	0.54 [0.50–0.58]
Q3 (0.31–0.43, N = 547)	0.87 [0.84–0.90]	0.64 [0.60–0.68]
Q4 (0.43–0.83, N = 546)	0.83 [0.79–0.86]	0.73 [0.70–0.77]
Number of carriers offering plans in the county		
1 carrier (N = 207)	0.32 [0.26–0.38]	0.17 [0.12–0.22]
2-4 carriers (N = 1,728)	0.85 [0.83–0.86]	0.57 [0.55–0.60]
5+ carriers (N = 252)	0.97 [0.95–0.99]	0.83 [0.79–0.88]
Medicaid expansion status		
Expansion state	0.73 [0.70–0.76]	0.42 [0.39–0.45]
Non-expansion state	0.87 [0.85–0.88]	0.66 [0.64–0.69]

© 2022 Kong E et al. *JAMA Health Forum.*

Characteristics associated with affected counties

Bivariate regression results

We use bivariate regression to assess features associated with counties experiencing turnover in zero-premium silver plans. Below, we consider county shares of self-reported race (white, non-white, black), and ethnicity (Hispanic, non-Hispanic), and the share of enrollees in the 100-150% FPL income group.

We consider two outcome variables. Our first outcome is a binary variable for whether a county has at least one zero-premium plan in 2021 that has a positive premium in 2022. Our second outcome is a binary variable for whether all zero-premium plans in a county have a positive premium in 2022.

We restrict to counties with at least one zero-premium plan in 2021. All regressions are weighted by the number of enrollees in the 100—150% FPL income group and use robust standard errors. We report full regression results below (**eTable 3**).

eTable 3: Bivariate association between zero-premium plan turnover and county race/ethnicity

A. At least one zero-premium plan in 2021 becomes a positive-premium plan in 2022

	(1)	(2)	(3)	(4)	(5)	(6)
White	-0.290***					
	(0.0304)					
Non-white		0.290***				
		(0.0304)				
Black			-0.142			
			(0.114)			
Non-Hispanic				-0.160***		
				(0.0269)		
Hispanic					0.195***	
					(0.0354)	
100-150% FPL						0.244***
						(0.0323)
Constant	1.060***	0.770***	0.972***	1.040***	0.929***	0.832***
	(0.00972)	(0.0220)	(0.0112)	(0.0152)	(0.00928)	(0.0185)
Observations	2,187	2,187	1,524	2,187	1,350	2,187

Robust standard errors in parentheses
*** p<0.01, ** p<0.05, * p<0.1

© 2022 Kong E et al. *JAMA Health Forum.*

B. All zero-premium plans in 2021 become positive-premium plans in 2022

	(1)	(2)	(3)	(4)	(5)	(6)
White	-0.735***					
	(0.0548)					
Non-white		0.735***				
		(0.0548)				
Black			0.178			
			(0.233)			
Non-Hispanic				-0.343***		
				(0.0484)		
Hispanic					0.487***	
					(0.0924)	
100-150% FPL						0.666***
						(0.0658)
Constant	1.125***	0.390***	0.861***	1.042***	0.789***	0.522***
	(0.0227)	(0.0418)	(0.0379)	(0.0349)	(0.0231)	(0.0409)
Observations	2,187	2,187	1,524	2,187	1,350	2,187

Robust standard errors in parentheses
*** p<0.01, ** p<0.05, * p<0.1

© 2022 Kong E et al. *JAMA Health Forum.*

Results by quartiles of each characteristic

The relationship between the probability that one or all zero-premium plans become positive-premium plans and each characteristic above may not be linear (this is the case, in particular, for the share of black enrollees in each county). Below, we report results from regressions of the outcome on quartiles of each characteristic (**eTable 4**). Regressions omit the constant term to allow inference for all four quartiles without an omitted quartile. Independent variables are shown in each column heading.

eTable 4: Zero-premium plan turnover by quartile of county race/ethnicity share

A. At least one zero-premium plan in 2021 becomes a positive-premium plan in 2022

	(1)<br>White	(2)<br>Non-white	(3)<br>Black	(4)<br>Non-Hispanic	(5)<br>Hispanic	(6)<br>100-150% FPL
Quartile 1	0.980***	0.724***	0.806***	0.981***	0.883***	0.819***
	(0.00571)	(0.0393)	(0.0338)	(0.00560)	(0.0220)	(0.0339)
Quartile 2	0.931***	0.840***	0.938***	0.971***	0.884***	0.873***
	(0.0124)	(0.0272)	(0.0148)	(0.00549)	(0.0241)	(0.0260)
Quartile 3	0.840***	0.931***	0.975***	0.842***	0.935***	0.940***
	(0.0273)	(0.0124)	(0.00791)	(0.0254)	(0.0138)	(0.0112)
Quartile 4	0.724***	0.980***	0.952***	0.657***	0.986***	0.976***
	(0.0393)	(0.00572)	(0.0162)	(0.0503)	(0.00492)	(0.00677)
Observations	2,187	2,187	1,524	2,187	1,350	2,187

Robust standard errors in parentheses
*** p<0.01, ** p<0.05, * p<0.1

B. All zero-premium plans in 2021 become positive-premium plans in 2022

	(1)<br>White	(2)<br>Non-white	(3)<br>Black	(4)<br>Non-Hispanic	(5)<br>Hispanic	(6)<br>100-150% FPL
Quartile 1	0.922***	0.447***	0.570***	0.908***	0.750***	0.583***
	(0.0250)	(0.0412)	(0.0566)	(0.0284)	(0.0349)	(0.0633)
Quartile 2	0.751***	0.623***	0.822***	0.868***	0.745***	0.636***
	(0.0354)	(0.0393)	(0.0343)	(0.0235)	(0.0375)	(0.0521)
Quartile 3	0.622***	0.751***	0.885***	0.660***	0.787***	0.796***
	(0.0394)	(0.0354)	(0.0382)	(0.0392)	(0.0353)	(0.0309)
Quartile 4	0.448***	0.922***	0.903***	0.389***	0.921***	0.916***
	(0.0412)	(0.0250)	(0.0323)	(0.0570)	(0.0285)	(0.0270)
Observations	2,187	2,187	1,524	2,187	1,350	2,187

Robust standard errors in parentheses
*** p<0.01, ** p<0.05, * p<0.1

© 2022 Kong E et al. *JAMA Health Forum.*

Additional analysis: changes in carrier and plan counts

Below, we relate the likelihood that a county is affected (i.e., has a zero-premium plan become a positive-premium plan) to changes in either the number of carriers (i.e., the number of distinct insurers) or the number of plans (each insurer may offer multiple plans). For the analyses that follow, we limit to the 2,187 out of 2,449 counties with enrollment data for the 100—150% FPL income group and at least one $0 silver plan in 2021.

Carrier entry

We find that 56% of these N=2,187 counties experience no change in the number of carriers (synonymous with "issuers"). Among the remaining counties, the majority gain 1 or 2 carriers (27% and 11% of all counties respectively, see **eFigure 1, Panel A** below). Very few counties (N=10) experience a decline in the number of carriers by 1.

We find that counties gaining carriers are almost certain to have at least one zero-premium plan become positive-premium, whereas counties with no change in the number of carriers (or a drop in the number of carriers) are less likely to be affected (see binscatter below, **eFigure 1, Panel B**).

eFigure 1: Turnover in zero-premium plans is associated with changes in the number of carriers

A. Change in the number of carriers per county ***B. Turnover is associated with carrier entry***

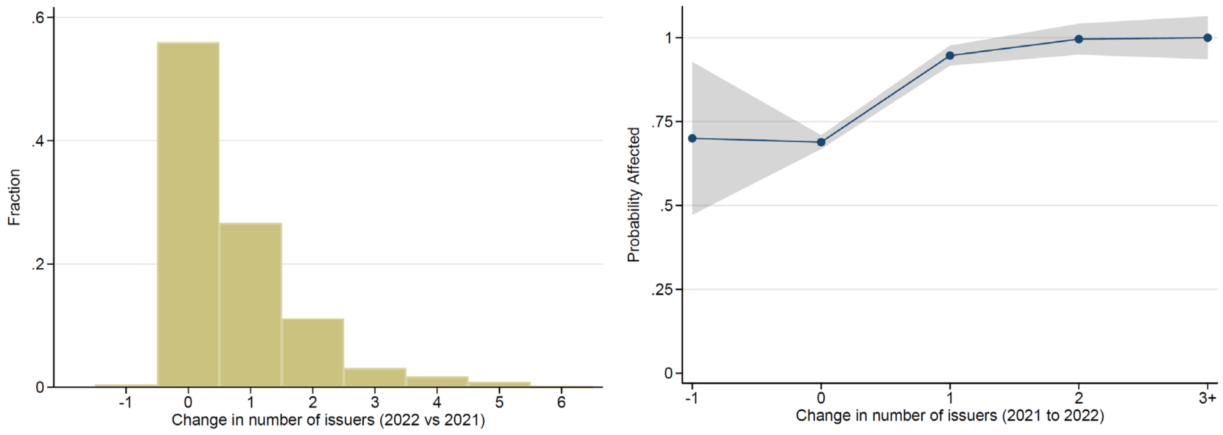

NOTES: Histogram (Panel A) shows the change in number of carriers from 2021 to 2022 for each county. Binned scatterplot (Panel B) relates the change in number of carriers to the probability that a county has at least one zero-premium plan become a positive premium plan from 2021 to 2022. Counties adding 3 or more carriers are combined. Shaded area denotes 95% confidence interval based on robust standard errors.

© 2022 Kong E et al. *JAMA Health Forum.*

Plan entry

We repeated the analysis above with plan entry rather than carrier entry. As shown in the histogram below, many counties (21%) experience no change in plans, but the majority of counties experience plan entry between 2021—2022 (70% of counties, **eFigure 2, Panel A**). Only 9% of counties experience a decline in plans. The binscatter below reproduces Figure 2 in the main text and shows that counties with no change in the number of plans are least likely to be affected, whereas counties with increases *or* decreases in the number of plans tend to see turnover in the set of zero-premium plans (**eFigure 2, Panel B**). The ~35% of counties that add 5 or more plans are virtually guaranteed to be affected by turnover in zero-premium plans.

eFigure 2: Turnover in zero-premium plans is associated with changes in the number of plans

A. Change in the number of plans per county ***B. Turnover is associated with plan entry and exit***

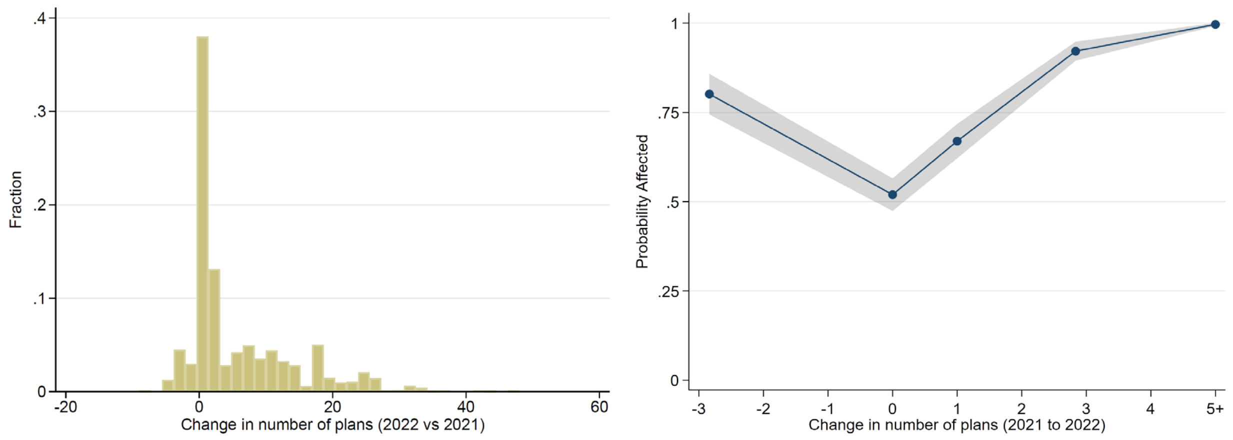

NOTES: Histogram (Panel A) shows the change in number of plans from 2021 to 2022 for each county. Binned scatterplot (Panel B) relates the change in number of plans to the probability that a county has at least one zero-premium plan become a positive premium plan from 2021 to 2022. Points in the binned scatterplot represent within-bin averages for counties adding between -9 and -1 plans (average = -3), 0 plans, 1 plan, or 2—4 plans. A separate point at x = 5 represents counties adding 5 or more plans. Shaded area denotes 95% confidence interval based on robust standard errors.

© 2022 Kong E et al. *JAMA Health Forum.*

Additional analysis: carrier and plan counts in 2021

The number of carriers (or plans) in each county in 2021 is also predictive of whether a county experiences turnover in at least one zero-premium plan. Below, we report histograms and binned scatterplots that are analogous to the results in the previous section on carrier/plan entry.

We find that counties with more carriers or plans in 2021 were more likely to have a zero-premium plan in 2021 become a positive-premium plan in 2022 (**eFigure 3**).

eFigure 3: Turnover in zero-premium plans is associated with the number of carriers in 2021

A. Number of carriers per county in 2021

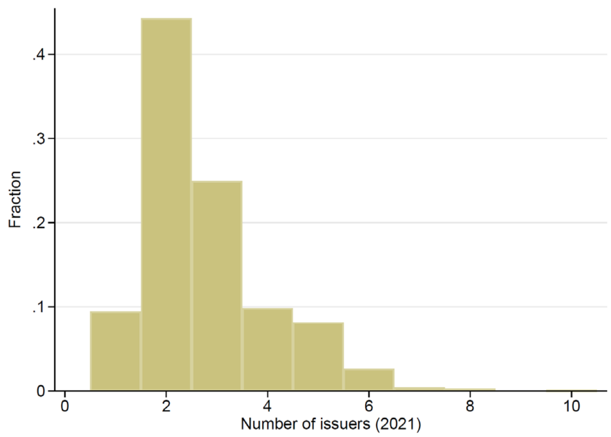

B. Turnover is associated with number of carriers

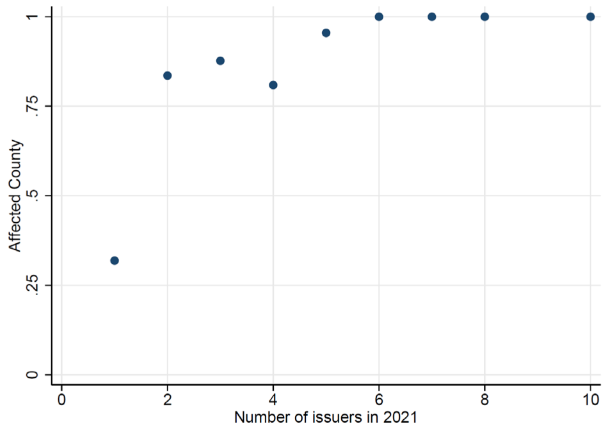

C. Number of plans per county in 2021

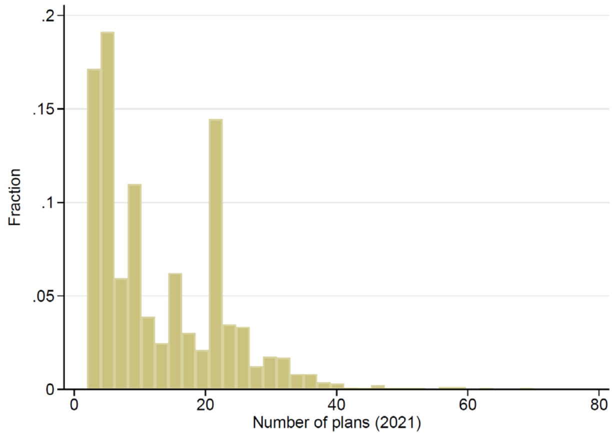

D. Turnover is associated with number of plans

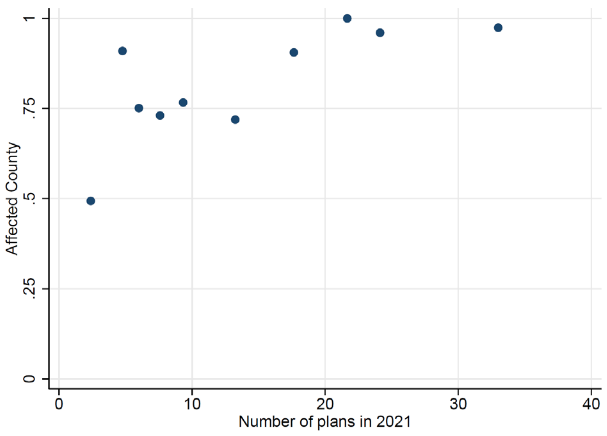

NOTES: Histograms show the number of carriers (Panels A) or plans (Panel C) in each county in 2021. Binned scatterplots show the relationship between the number of carriers in 2021 (Panel B) or the number of plans in 2021 (Panel D) and the probability that a county experienced turnover in at least one zero-premium plan from 2021 to 2022.

© 2022 Kong E et al. *JAMA Health Forum.*
